# Performance Evaluation of a Novel Digital Flow-Imaging IV Infusion Device

**DOI:** 10.1109/OJEMB.2025.3641824

**Published:** 2025-12-08

**Authors:** Robert D. Butterfield, Nathaniel M. Sims

**Affiliations:** RDB Consulting Poway CA 92064 USA; Massachusetts General Hospital 2348 Boston MA 02114 USA

**Keywords:** Coefficient of variation (CV), computational simulation, flow-imaging infusion device, legacy infusion pump (LIP), standards-based performance evaluation

## Abstract

*Goal*: Assess performance and potential use of a novel, servo-controlled, gravity-driven infusion device with FDA regulatory clearance obtained 3/1/2024(K242693). *Introduction:* "*SAFEflow^TM^*" (SF) using real time flow measurement and feedback control, has been cleared by USFDA. We hypothesized that due to its architecture using video imaging there will be both benefits, and functional contrasts with the behavior of legacy infusion pumps (LIPs). *Methods:* We conducted type-tests of critical metrics using AAMI and IEC standards together with computational simulations. Results were compared with claimed and measured performance of two widely-used LIPs. *Results/Discussion:* Across its rated flow range of 1-600 ml h^−1^, SF’s measured *flow* performance was superior (95/95% confidence/reliability −4.3% to +4.5% mean flow rate accuracy) to claims of two LIP designs, Occlusion detection was more consistent and rapid across flow rates and requiring less user interaction. *Conclusion:* SF’s infusion performance was superior with reduced weight, size, and low parts count compared to LIPs. Regulatory evaluation standards may require updating for this class of infusion device.

## I. Introduction

Transformation of life-critical intravenous medical fluid and drug administration to fully implement modern digital technologies has been slow in coming to this field for many reasons including: lack of effective competition, regulatory framework dependent on predicate device citation, and the inertia of a large installed base.

**Fig. 1.**
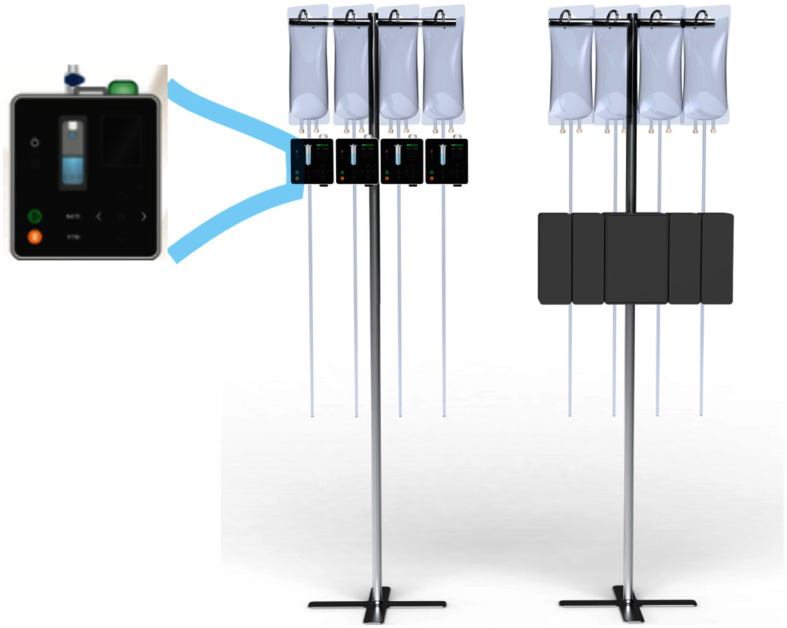
Comparison of four infusion channels using *SF* (left) and typical size *LIP* (right).

**Fig. 2:**
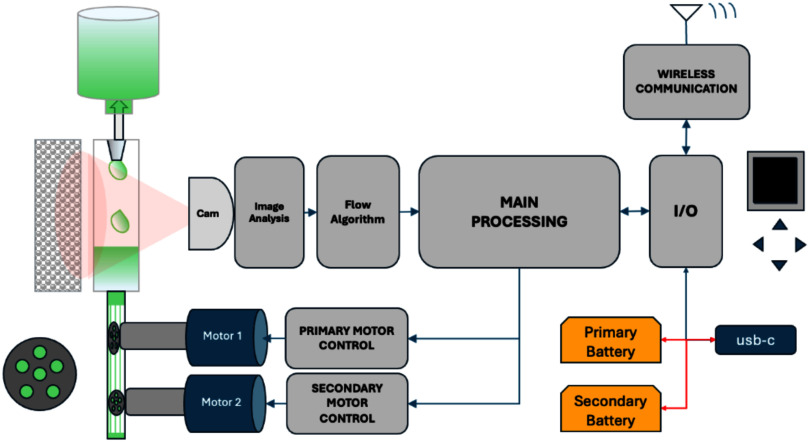
Conceptual block diagram.

While gravity-driven IV controllers were commonly used decades ago, their ability to infuse was limited by simplistic drip-rate sensors, and non-linear flow rate restricting elements. The SF design employs video-imaging-based flow-sensing and a novel multi-lumen fluid-restriction element, offering increased accuracy and fault detection performance as well as reliability, safety, small size and lower cost (Figs. 1 and 2).

We hypothesized that SF would perform at least as well as contemporary LIP designs over its intended flow rate range. To test this, we reviewed manufacturer claims and carried out a series of laboratory “type tests” using six SF devices and six LIPs of two different designs. Special attention was focused on performance issues unique to a gravity-propulsion, servo-controlled infuser, such as flow-rate-range limitations, and potential flow instability due to varying outlet pressures that may occur during titration and co-infusion.

## II. Materials and Methods

System architecture was reviewed with consideration of design topics including: safety, reliability, power source(s), flow sensing, feedback control, size and weight. See Supplementary Materials (SM) section I Introduction.

To explore flow capabilities of the SF controller, a set of catheter dimensions [3], [4], [5], [6] and vascular resistance [7] were used to calculate fluid resistance and maximum available flow at controller head heights (distance from controller to patient level) (HH) from 50 to 150 cm (Tables SM-IV and SM-V).

Fluid delivery performance of SF was measured gravimetrically on six sample units with frequently-used adult and infant catheters across flow rates from 1-600 ml h^−1^ (Fig. SM-3 and Table SM-2). Test methods adopted from AAMI TIR101 [1] combined with the "stair-step" flow protocol (Fig. SM-4) by Knudsen [8], were applied for tests which lasted from 20 to 23 hours. These provided data for analysis of mean, short-term and transition rate performance.

Mean Flow Rate Accuracy (MFRA) and its sensitivity to variation of inlet and outlet pressures, at 25 ml h^−1^ over 1 hour of infusion was measured in six SF controllers and six LIPs of two widely-used designs.

Short-Term Flow Accuracy (STFA) measurements of both SF and LIPs used flow data at 1 ml h^−1^ collected under nominal inlet/outlet pressure conditions for at least 4 hours.

Co-infusion through a common catheter using two SF devices is shown in Fig. SM-3b and Table SM-I. Numerical simulation guided choice of catheter fluid resistance and flow rates for evaluation of flow accuracy and stability(SM Appendix A3).

Occlusion detection response time was measured for SF devices using 100 cmH_2_O head height (HH) at five flow rates from 1 to 600 ml h^−1^. The LIP’s response was measured at low, mid and high sensitivity settings with administration set outlet level with the pump. At each flow rate and each setting, the patient end of the administration set was occluded and time to alarm measured by stopwatch.

## III. / IV. Results / Discussion

Refer also to SUPPLEMENTARY MATERIAL for additional details and data.

### A. Flow Range Capability

The SF device tested had an upper flow rate limit of 600 ml h^−1^ bounded by its current video imaging flow sensor and nominal 50 μL drop forming orifice. Calculated flow capability (Table SM-III) at SF’s nominal head-height of 100 cmH_2_O using adult catheters with fluid resistance of 0.15 cmH_2_O h ml^−1^ or less was at least 600 ml h^−1^. With catheters common for premature infants such as Luther 1.2F x 8 cm, fluid resistance limited maximum flow rate to 2-3 ml h^−1^. However, even very small-bore catheters such as the Luther 1.9F x 30 cm provide an estimated upper flow limit of 63 ml h^−1^, well above most patient’s requirements.

### B. Mean and Short-Term Flow Accuracy/Variability and Sensitivity of Accuracy to Pressure and HH

Mean flow accuracy of SF over flow rates measured from 1 to 600 ml h^−1^ ranged from −1.3 to +1% with 95%/95% confidence/reliability limits of −4.3% and +4.5% respectively [9] for all flow rates pooled. This at least meets or exceeds claims of LIP’s (Table SM-IV) [9], [10], [11].

Co-infusion was tested using two pairs of SF’s infusing through a 150 cm 5 g catheter (see SM section IIA). One SF ran at 38 ml h^−1^ while the second executed a stair-step pattern from 16 to 1 and back to 16 ml h^−1^. The mean error in the combined flows at each step ranged from −0.7% to 2.7%. These results demonstrate that SF devices can co-infuse with acceptable accuracy.

Due to SF’s use of continuous flow measurement in a feedback control loop, the impact of varying head-height on mean flow accuracy was small at −0.85% for each 50 cm HH reduction. In contrast, LIP’s tested exhibited overall between −3.2% to 5.2% (combined inlet and outlet) error per 50 cmH_2_O HH (Table SM-IV).

### C. Response to Flow Rate Programming Changes

Flow transition latency measures the *effective delay* per AAMI TIR101-2021 [1] in volume delivery when programmed flow rate is initiated or is adjusted, such as during clinical titration-to-effect of short half-life medications. In some designs, an initial acceleration may cause the delay to be negative, signifying an "advance" of the flow relative to the programmed delivery.

Initial flow following loading of a new SF administration set, produced a delivery *advance* of ∼2 minutes at 1 ml h^−1^ due to operation of the closed-loop control algorithm, (Fig. SM-6). All other programmed transitions, whether rising or falling, were within $ \pm $2 minutes, diminishing at higher flow rates.

While we did not test this characteristic in the LIP’s, the direct coupled mechanical design of these pumps produces nearly instantaneous translation of motor speed to flow.

### D. Occlusion Detection Response Time

**Fig. 3.**
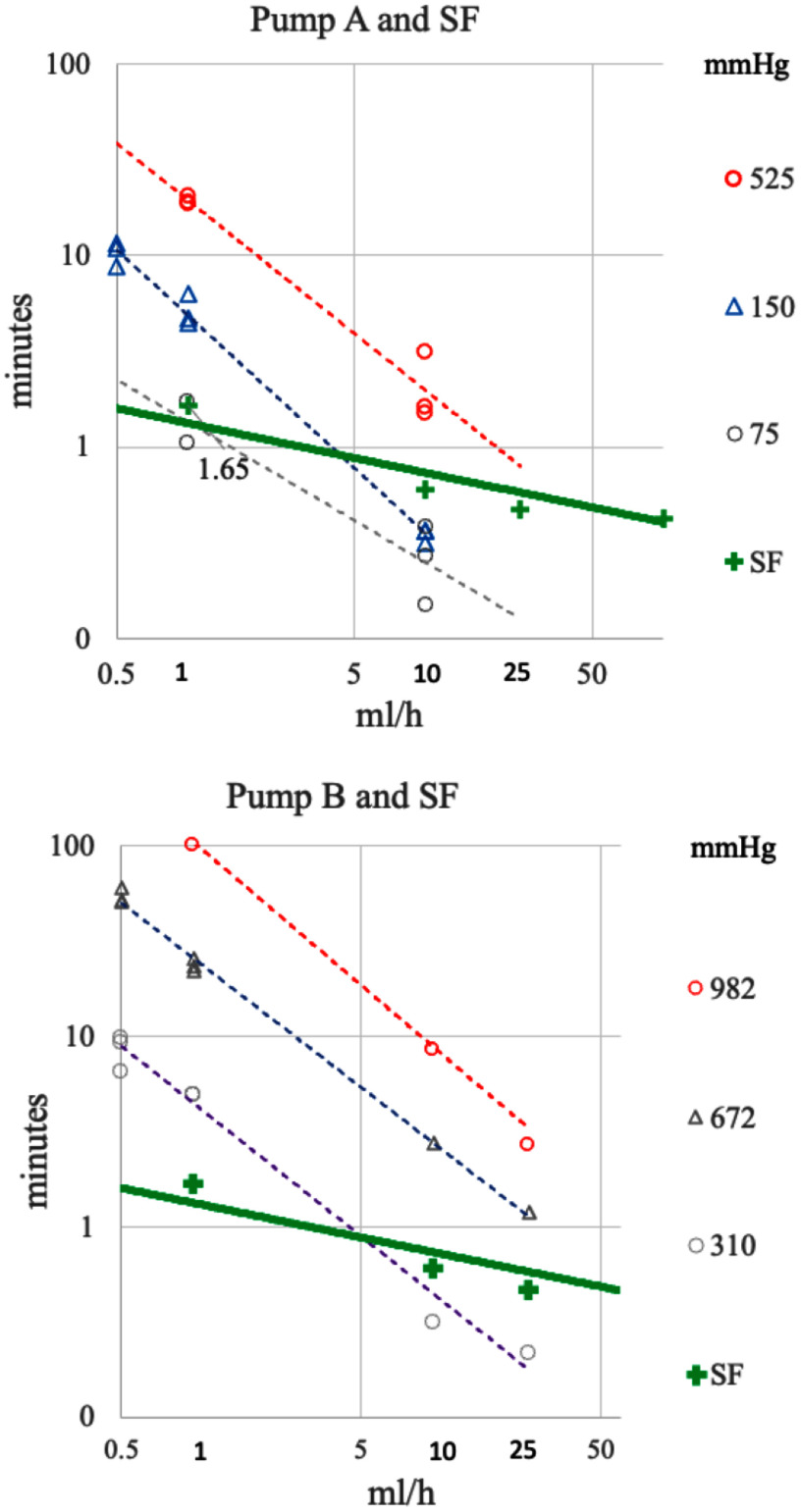
Comparison of measured time to detect occlusion vs flow rate and limit settings for two exemplary LIPs vs SF. Occlusion detection times are shown in log(time) vs log(flow rate).

Occlusion detection times of two LIP designs and SF were measured over a range of flow rates from 0.5 to 25 ml h^−1^ (Fig. 3). The LIPs were set to their low, mid and highest sensitivities. SF required no occlusion adjustments and was operated with 100 cmH_2_O nominal HH. LIPs using pressure measurement for occlusion detection have a wide range of settings. For example, pump "B" has a low sensitivity (longest response) limit of 982 mmHg, while pump "A" has a high sensitivity (shortest response) at a setting of 75 mmHg. Use of very high sensitivity settings often causes false alarms, especially at higher flow rates leading clinicians to reduce occlusion sensitivity, with the unintended consequence of undesirably long detection time at low flow rates and potential large bolus releases at high pressure [12].

SF produced shorter and significantly less variable occlusion times compared to LIPs. The occlusion limit setting requirement for LIPs directly affects both pressure at alarm and detection time while, for SF, direct flow measurement-based detection is independent of pressure as well as inherently limited to a low value.

### E. Comparison of SF With Commonly-Used LIPs

**Fig. 4.**
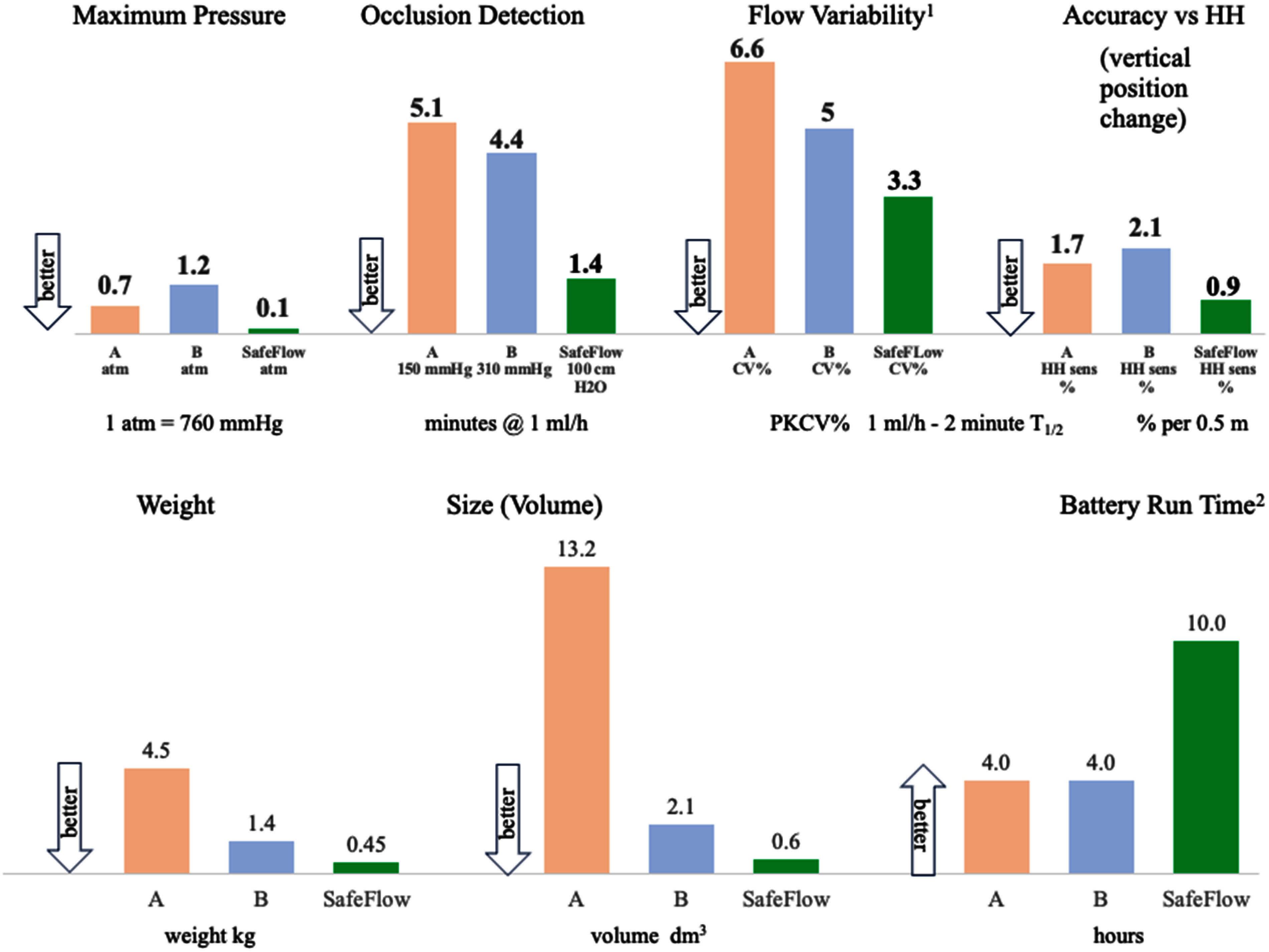
Comparison of key performance and architectural features of SF and LIPs. NOTE1. CV% values are averages of three pump runs over 3 hours NOTE2. Longer run time is preferred.

Table I compares capabilities of SF with published and sample measurements of two LIPs. Fig. 4 graphically depicts, in bar chart format, the categories of: flow accuracy, short-term flow variability, accuracy sensitivity to HH, maximum occlusion pressure, size, weight, and battery run time.

**TABLE I:** Comparison of Performance and Design Aspects of SF With Two Commonly Used LIPs

**Characteristic**	**SF**	**LVP A**	**LVP B**
Flow Range (ml h^−1^)	1-600	0.1-999.9	0.5-999.9
Flow Rate Accuracy (Claimed, Measured)	+1.1% / -1.3% (mean range) -4.3 / 4.5 (95%/95% confidence/ reliability)	Claimed: ±5% "Standard Conditions" Measured: +1.5%	Claimed: ±5% DEHP tube ±10% non-DEHP tube "Standard Conditions" Measured: +5.1%
Max Infusion Time (h)	72	96	96
			
Measured Sensitivity of MFRA to 0.5 m inlet head reduction	-0.85% (per 0.5m)	-2.5% (per 0.5m)	-4% (per 0.5m)
Measured Sensitivity of MFRA of 0.5 m outlet head increase			
		-0.72% (per 0.5m)	-1.2% (per 0.5m)
Measured Impact on MFRA of reduced inlet pressure allowed by device		-12.5% Flow Reduction	-17.6% Flow Reduction
Measured Impact MFRA of 10 psi outlet	N/A (occlusion detected at HH pressure)	-7.5% Flow Reduction	-12% Flow Reduction
			
Measured Short-Term Flow Accuracy (CV%) at1 ml h^−1^	2.5 - 4%	4.5 - 10%	4 - 6%
		
Occlusion Time (mins) at 1 ml h^−1^ [threshold mmHg]	1.3	1.4, 4.9, 19 [75, 150, 525]	4.4, 25, 107 [310, 672, 982]
Maximum pressure (cmH_2_O)	150	714	1224
			
Battery Life (h)	8 (claimed) 10 (max at 125 ml h^−1^)	4	4 (@125 ml h^−1^)
Weight (kg)	0.45	4.95	1.625
Power Cord	OPTIONAL	YES	YES (AC/DC adapter)

SF achieved at-least equivalent or superior performance to LIPs using a design offering inherent safety improvements and ergonomic advantages due to reduced size/weight. Moreover, the opportunity for substantial reduction in cost is inherent in its lower complexity and parts count.

Benefits of small-size, remote monitoring and control are discussed in SM IV*-F* and *-G*. Suggested design enhancements are provided in SM Section IV*-H*.

## V. Conclusion

In both design including: size, weight, low parts count and performance (including: flow accuracy, rapid and low-pressure occlusion detection, and transition of rates) we found the SF design at least "non-inferior" to evaluated legacy infusion pumps. Available data indicate SF’s unique design is able to exceed critical performance requirements while reducing weight, size and offering advanced programming safety features.

Flow rate range, at both extreme low and high rates may be expanded by implementation of delivery sets with larger and/or smaller drip-forming-orifice(s).

We note that formal testing standards for infusion devices will need expansion to embrace the unique design and operational features of this new class of infusion devices. Metrics such as flow transition-delay should be incorporated to disclose performance under clinical conditions including initiation of flow and periodic titration.

## Supplementary Materials

Supplementary Materials
