# Supplementary material for: Performance Evaluation of a Novel Digital Flow-Imaging IV Infusion Device
Source: IEEE Open J Eng Med Biol. 2025 Dec 8;7:43–6. doi: 10.1109/OJEMB.2025.3641824 (PMC12885485; doi:10.1109/OJEMB.2025.3641824)
Supplement: Supplementary Materials [file supp1-3641824.pdf]

Performance Evaluation of a Novel digital Flow-Imaging IV Infusion Device[1]

Robert D. Butterfield, *Member, IEEE*[2] and Nathaniel M. Sims, *Member, IEEE*[3]

I. INTRODUCTION

These Supplemental Materials (SM) expand on topics in the associated Technical Note (N), providing additional narrative, data, plots and diagrams.

***Index Terms*— (CV) coefficient of variation, computational simulation, flow-imaging infusion device, (LIP) legacy infusion pump, standards-based performance evaluation.**

Problem Statement

Many *"legacy infusion pump"* (LIP) designs in common use have well-recognized weaknesses and limitations including those following [1] [2]. We explored the hypothesis that a gravity-propelled controller, employing feedback control based on video flow imaging, could effectively address some or all of these concerns.

Performance
- Fluid Delivery Accuracy
- Slow Detection of Occlusions
- Delayed Flow Onset and Transition

Patient Injury Risks:
- Uncontrolled Flow
- Air Embolus
- Non-Detection of Occlusion
- Tissue Damage
- False Alarms
- Programming Errors

Usability
- Size/Weight
- Battery Life - Uncertainty
- Vulnerability to Damage

Expanding several of the most vital of these-

A. Fluid delivery accuracy of many LIPs is adversely impacted by variable inlet and outlet pressures, especially when operating outside manufacturer-defined conditions [3] [4]. Increasing inlet pressure can raise flow rate by expansion of the tubing under control, while decreasing inlet pressure reduces flow. Similarly, elevated outlet pressure may reduce flow, and negative outlet pressure may undetectably increase flow above specified accuracy. These pressure variations may silently alter volume delivery resulting in challenges to titration of rapid-acting medications as well as requiring "workarounds" to ensure volume over time accuracy is achieved to comply with electronic medication record orders (EMAR). [5]

B. Uncontrolled free flow reported in the MAUDE (Manufacturer And User facility Design Experience) database is unacceptably frequent and may lead to severe patient harm or death. [6]

C. Delayed commencement and transition of flow, especially at low rates, may pose challenges to successful titration of rapid-acting, life-critical medications [7] [8].

E. Delayed detection of flow interruptions due to occlusion of either inlet or outlet create special risks for delivery of rapid-acting medications, where even brief interruption can have serious patient impact [9].

F. Frequent false alarms due to their inability to directly measure fluid flow has produced "alarm fatigue" and led to a 'fail-stop' design philosophy with potential patient harm due to loss of drug effect, as reported in the MAUDE database [6] and manufacturer recalls.

Architecture of the recently cleared (**K232316**) [10] SAFEflow™ (SF) controller intends to remedy many of these weaknesses (see Fig. SM-2).

A. High-Reliability Flow Control Design
Flow rate is controlled by varying compression of a multi-lumen silicon rubber segment located just below the drip chamber inside a dedicated administration set. A pair of independently powered linear actuators adjust and monitor forces on the tubing providing redundant ability to regulate or stop flow should a fault conditions arise.

B. Cord-free operation / Battery Power
Battery life of (10+ hours at 125 ml h^{-1}) permits cord-free operation for many therapies. For longer-term infusions

[1] This work received financial support from DEKA Research Corporation
[2]RDB is an AAMI Fellow, a member of the AAMI Infusion Device Standards Committee, IEC TC62D/MT23 Committee, a principal at RDB Consulting, Poway, CA, USA, and was for 38 years a research engineer at Becton Dickinson involved in infusion pump design and testing. (correspondence email:robert.dwaine.butterfield@gmail.com)

[3] NMS is Physician Advisor, Biomedical Engineering, Massachusetts General Hospital, non-industry co-chair, AAMI Infusion Device Committee, and Newbower/Eitan MGH Endowed Chair in Biomedical Technology Innovation (nsims@mgh.harvard.edu.)

power is supplied via a USB data/cable charging connector. Low power demands permit the convenience and safety of portable operation adequate for many therapies.

C. Flow / Fluid State Sensing
Continuous monitoring of the formation and movement of drops together with drip chamber fill level make possible elevation-independent flow accuracy and rapid detection of occlusions and faults, even at the lowest flow rates.

D. Construction
Low weight and size, on the order of 10% of conventional IV pumps, was achieved by use of high-efficiency batteries, circuitry and miniaturized motor/gear assemblies. An impact-resistant case, IPX-42 (Ingress Protection) rating and assembly, is designed and tested to withstand predictable impacts from falls or collision during transport.

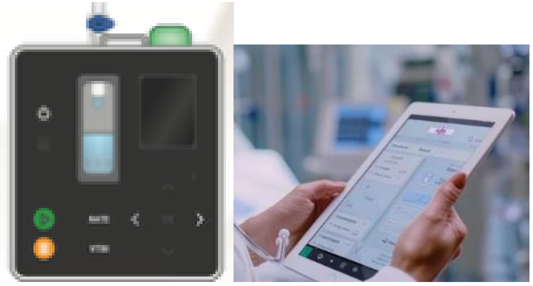

Fig. SM-1. SF communicating wirelessly with tablet computer.

E. Wireless/Wired Communication
Wireless communication technologies include Wi-Fi and Bluetooth in addition to a wired USB-C data/power port. A secure digital radio link is designed to assure a high uptime ability, making it possible for cloud-based applications to perform conventional dose error reduction and directed order processes as well as remote clinician monitoring and control via a smartphone or tablet.

F. Remote User Interface
User interaction with SF will be augmented by wireless remote monitoring and control using a tablet computer (Fig. SM-1) based on the insulin pump technology platform cleared by USFDA (K213536) [10]. Multiple interface designs adapted to specific care regimens are thereby possible. Use of tablets will provide a low cost and flexible platform for automatic data capture (i.e. bar code or RFID) for incorporation of BCMA (barcode medication administration) and EMAR (electronic medical record) data into clinical workflows.

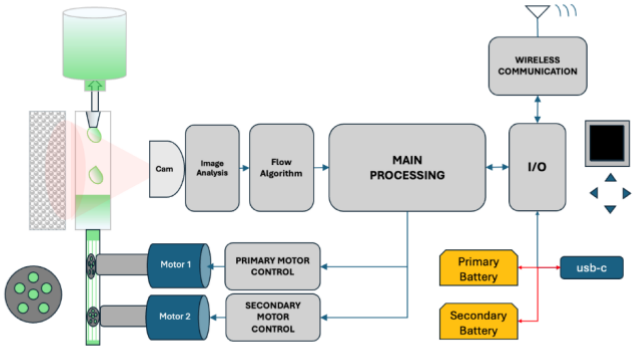

Fig. SM-2. SF Conceptual block diagram

II. MATERIALS AND METHODS

Six SF devices evaluated were physically identical to the cleared K232316 design with addition of ability to operate over an extended range from 1 to 600 ml h^{-1} using software expanding lowest flow rate to 1 ml h^{-1}. Performance test protocols were designed based on recognized test methods (AAMI TIR101 Technical Information Report) [11], IEC60601-2-24 [12] and Knudsen [8]). Gravimetric testing was conducted on the six SF devices, and selected tests were performed on six LIPs from two manufacturers (i.e. a 3 of each).

A. Flow Rate Capability and Performance
We estimated SF's maximum potential flow with different available catheters by compiling a table of catheter dimensions and flow resistance and computing the achievable flow rates at each of four potential head-heights (HH) (Tables SM I-III).

We measured SF's mean flow rate accuracy (MFRA) by recording gravimetric flows over two ranges of flow (1-16 and 10-600 ml h^{-1} using a "stair-step" [8] flow protocol over 20-23 hours (Fig. SM3-a, SM-4, Table SM-II)). The 10-600 ml h^{-1} range, typical for adult infusions, was measured at three nominal head-height (HH) pressures of 50,100, and 150 cmH_2O infusing through a low fluid resistance (<0.04 cmH_2O h ml^{-1}) cannula representing commonly used adult catheters.

For both SF and LIPs, we recorded gravimetric flow at 25 ml h^{-1} with variations of inlet and outlet hydrostatic pressures allowing calculation of sensitivity of MFRA to inlet and outlet pressure. We also recorded gravimetric flow at 1 ml h^{-1} under nominal inlet/outlet pressure conditions to compute MFRA and short-term flow accuracy (STFA) using the PK-CV method of TIR101 [11].

Some patients, particularly children, require long, narrow bore central catheters for continuous low-rate infusions. These catheters present high resistance to flow, which will constrain the maximum flow rate. Accordingly, to explore SF performance in the 1-16 ml h^{-1} range, we measured flow with and without an inline 3.75 cmH_2O-h-ml^{-1} restrictor, approximating the resistance of a 30 cm long Vygon

Epicutaneo Cava (Si) 2Fr/24g [13]. This tested SF's ability to maintain flow stability when considerable pressure loss occurs at flow rates, such as 16 ml h^{-1}, that might be the maximum expected when giving parenteral nutrition to a child.

We evaluated ability to co-infuse (Fig. SM-3b) from two devices using both a numerical computational simulation (Fig. SM-10) and a laboratory study in which a stair-step flow pattern (ranging from 1-16 ml h^{-1} was merged with a constant flow (38 ml h^{-1}) passing through a 1.17 cmH_2O h ml^{-1} fluid restrictor simulating resistance of a 1.9F x 20 cm peripherally inserted central catheter (PICC).

Flow Rate Range Performance Testing					
	**Flow Rates (ml h^{-1})**	**Patient type**	**Catheter resistance (cmH_2O h ml^{-1})**	**Clinical Usage**	**For comparison - exemplary similar Vascular Access Device (VAD)**
High-Rate Range	10-600	adult	0.04	fluid hydration/ resuscitation	Central Venous Catheter Arrow [12] ML-00703 0.0383 cmH_2O h ml^{-1}
Low-Rate Range	1-16	infant / child	3.75	lipid, TPN, carrier	PICC Catheter [11] Vygon Epicutaneo-Cava 0.6 x 0.3 mm x 30 cm 3.75 cmH_2O h ml^{-1}
Co-infusion via Common Volume	(#1)38 fixed (#2) 16-1-16 stair-step	critical care co-infusion	1.17	vasoactive	PICC Single Lumen [13] 1.9F x 20 cm 0.9 cmH_2O h ml^{-1}

Table SM-I. Summary of the tests conducted to determine SF performance in the presence of downstream inline resistance simulating several realistic clinical scenarios.

B. Flow Rate Change Response

A "stair-step" flow protocol, producing sequenced changes in programmed flow rate [8], was used with adaption of AAMI TIR101 [11] (Fig. SM-4) to gather data for calculation of flow transition delays.

C. Occlusion Detection

Occlusion detection response time (TTA) was measured for the SF devices using nominal HH of 100 cmH_2O at five flow rates from 1 to 600 ml h^{-1} by occluding the patient end of the delivery set and measuring time to alarm by stopwatch. We similarly measured two commercial LIP's and plotted results together on linear-log and log-log scales in Fig. SM-7. We plotted the power-fit equation of TTA vs flow rate for each device tested.

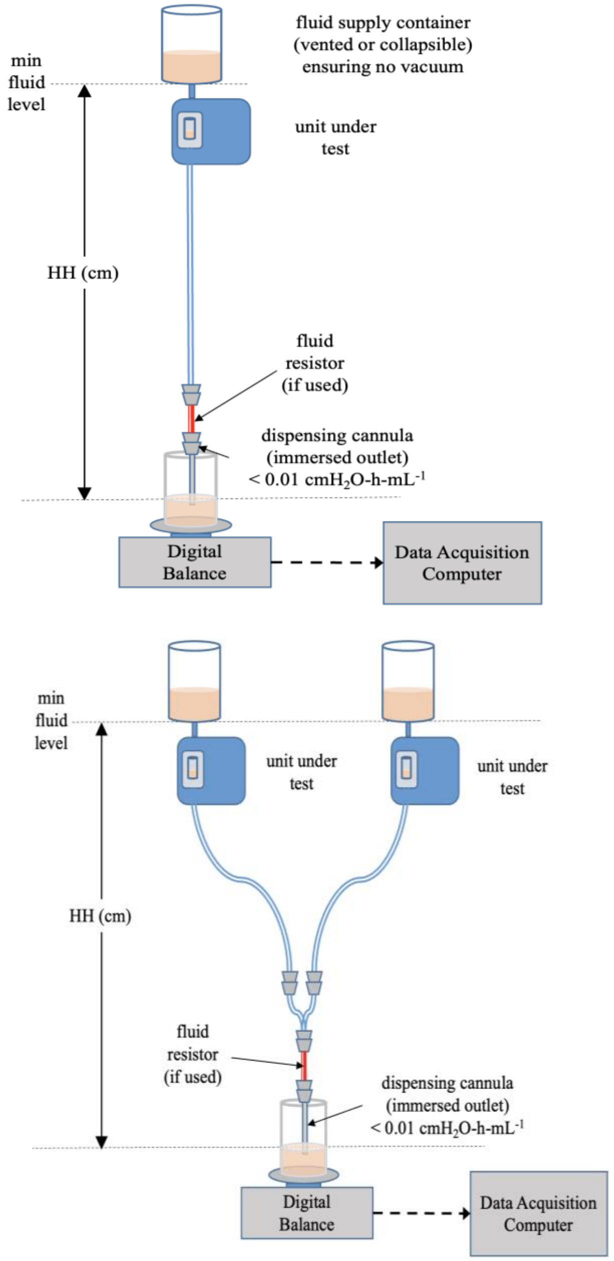

Fig. SM-3a. Test setups for single infusion (above), Fig SM-3b co-infusion (below).

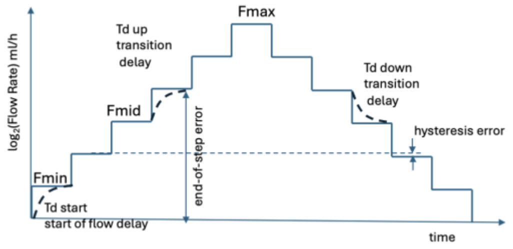

Fig. SM-4 Generic Stair-Step Flow Illustration

The "Stair-Step" flow protocol (Fig. SM-4) has been used previously by Knudsen et. al. [8] to characterize infusion device performance. It provides measurements of several aspects of fluid delivery including mean, short-term-flow accuracy and transition delays between successive set flow rates in a single test.

Low Range test (ml h^{-1})	Duration (h)		High Range test (ml h^{-1})	Duration (h)
1	3		10	2
2	3		20	2
4	2		40	2
8	2		80	2
16	2		150	1.25
8	2		300	0.75
4	2		600	0.75
2	3		150	1.25
1	3		80	2
			40	2
			20	2

Table SM-II Stair-Step Flow Protocol Test Rates and Durations

III. RESULTS

A. Flow Range Capability and Performance

Flow imaging enables continuous closed loop adjustment of regulating resistance allowing adaptation to widely varying HH and in-line flow resistances. Testing confirmed that when the programmed flow is not achievable due to e.g. insufficient head height, an alert is issued. Calculated maximum flow rates (see following Eq. SM-1) vs available expected head height (HH) for a range of commonly used catheters are shown in Table SM-III, columns c-e. This result includes the contributions of resistance of veins, delivery set and VAD using the formula:

$$\dot{Q}_{max} = P_{head}\,(R_v + R_{set} + R_{cath})^{-1} \quad \text{(SM-1)}$$

where:

$\dot{Q}_{max}$ is the flow rate in ml h^{-1};
R_v is the vein resistance [14];
R_{set} is the delivery set resistance;
R_{cath} the catheter resistance.

and

R = $8\mu L\pi^{-1}r^{-4}$ (SM-2) [7];
where:
L is length (m);
r is inner radius (m);
μ is dynamic viscosity (Poise or Pa-s).

Co-infusion was tested using two pairs of SF's, one infusing at a constant 38 ml h^{-1}, the second providing a stair-step flow sequence of 16, 8, 4, 2, 1, 2, 4 ,8, and 16 ml h^{-1}. The mean error in total flow was computed at each step. For pair 1, the mean error ranged from 0.9% to 2.7% while for pair 2, the mean error ranged from -0.7% to +1.8%. These results demonstrate ability of two SF devices to co-infuse accurately through a typical PICC catheter.

a	b	c	d	e
		Drive (Head) Pressure (cmH_2O)		
Catheter Fluid Resistance cmH_2O h mL^{-1}	Catheter (size length)	50	100	150
0.0004	AngioCath 14g x 2 in	600	600	600
0.001	AngioCath16g x 2 in	600	600	600
0.003	AngioCath18g x 2 in	600	600	600
0.008	AngioCath20 x 2 in	600	600	600
0.010	Luther PICCsingle5F x20 cm	600	600	600
0.010	BD Insyte24g x19mm	600	600	600
0.016	AngioCath22gx 1in	600	600	600
0.020	Luther PICCsingle5F x56 cm	600	600	600
0.020	BD Insyte.85 mmID x 180 cm	600	600	600
0.020	CVP Arrow 16g x 20 cm	600	600	600
0.033	AngioCath24g x 3/4 in	521	600	600
0.052	Spinal needles 20g x 3.5 in	435	600	600
0.057	Air Elim Filter Pall 0.22u	417	600	600
0.060	Luther PICCsingle3.5F x20 cm	407	600	600
0.080	Gesco PICC single 4F x 58 cm	350	600	600
0.090	Gesco PICCsingle5F x60 cm	327	600	600
0.150	Davol Silicone Broviac .7 mm ID x 73 cm	235	469	600
0.155	Gould 3 lumen Swan 7F x 133 cm	229	459	600
0.160	Gesco PICCsingle4F x60 cm	224	448	600
0.160	Gesco PICCsingle4F x60 cm	224	448	600
0.180	Luther PICCsingle3.5F x56 cm	206	412	600
0.180	Luther PICCsingle2.6 F x8 cm	206	412	600
0.230	Gesco PICC dual Distal 5F x 123 cm	171	341	512
0.290	Epidural Deseret 19g x 100 cm	142	283	425
0.347	Spinal needles 22g x 3.5 in	122	244	366
0.350	Gesco PICC single 3F x 60 cm	121	242	363
0.410	Luther PICCsingle1.9F x8 cm	106	211	317
0.450	Luther PICCsingle2.6F x20 cm	97	195	292
0.497	Epidural Encapsulon1200 19g x 100 cm	89	179	268
0.650	Gesco PICCsingle2F x28 cm	70	140	210
0.750	VygonEpiCutaneoCath0.6 x 0.3 mm 8 cm	62	123	185
0.840	Gesco PICCsingle3F x60 cm	55	111	166
0.840	Gesco PICCsingle3F x60 cm	55	111	166
0.959	Epidural Deseret 20 g x 100 cm	49	98	147
0.970	Luther PICCsingle1.9F x19 cm	48	97	145
1.000	Gesco PICC dual Proximal 5F x 123 cm	47	94	141
1.250	Luther PICCsingle2.6F x56 cm	38	76	114
1.314	Spinal needles 26g x 3.5 in	36	73	109
1.410	VygonEpiCutaneoCath0.6 x 0.3 mm 15 cm	34	68	102
1.520	Luther PICCsingle1.9F x30 cm	32	63	95
1.827	Epidural Travenol 19g x 100?	26	53	79
2.830	VygonEpiCutaneoCath0.6 x 0.3 mm 30 cm	17	35	52
24.990	Luther PICCsingle1.2F x8 cm	2	4	6
34.920	VygonEpiCutaneoCath0.6 x 0.3 mm 50 cm	1	3	4
43.740	Luther PICCsingle1.2F x14 cm	1	2	3
62.480	Luther PICCsingle1.2F x20 cm	-	2	2
78.110	Luther PICCsingle1.2F x25 cm	-	1	2

Table SM-III**.** Gravity propulsion calculated flow capability (ml h-1) vs Head Pressure (cmH_2O)) for clinically used catheters.

B. Mean and Short-Term Flow Accuracy/Variability and response to Varying head height

Fig. SM-5 illustrates typical LIP and SF flow patterns at 1 ml h^{-1}. Coefficient of Variation (CV T_{half} 2-minutes) for SF was 2.5%, while for LIPs A and B, CV was 10% and 6% respectively. A CV value less than 5% is considered desirable according to TIR101 [11].

MFRA of SF at flow rates from 1 to 600 mL h^{-1} (Table SM-II) ranged from -1.3% to +1%, confidence/reliability limits at 95%/95% were 4.3% and +4.5% (all flow rates pooled).

For SF, the reduction in delivered flow caused by reducing head-height was -0.85% per -0.5 m. In contrast, LIP's A and B flow was reduced by -2.5% and -4% per -0.5 m (reduction) on inlet, and 0.72% and 1.2% per +0.5 m (increase) on outlet (see Table SM-IV).

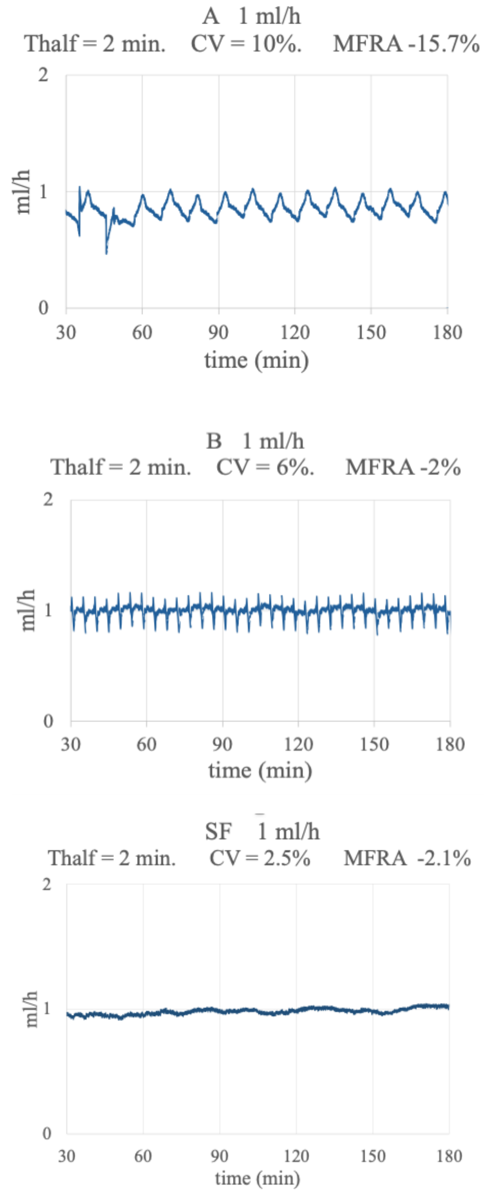

Fig. SM-5. Typical short-term flow variation of LIP A, B and SF at 1 ml h^{-1}. Measured data is low-pass filtered using a 2-minute half-life, 1 compartment PK model.
NOTE. Data was analyzed from 30 minutes onward to allow flows to reach steady state.

C. Response to *Flow Rate Programming Changes*
Flow transition latency per TIR101 [11] measures the *effective delay* in volume delivery when programmed flow rate is initiated or is adjusted such as during clinical titration-to-effect of short half-life medications. In some syringe pump designs, an initial deliberate acceleration may cause the delay to be negative signifying an "advance" of the flow relative to that programmed.

The SF devices at 1 ml h^{-1} produced an advance of slightly less than 2 minutes following loading due to operation of the control algorithm. Other transitions between flow rates, whether rising or falling, were well within ± 2 minutes, decreasing at higher flow rates shown in Fig. SM-6. We did not measure delay for the LIPs, however based on their mechanism design, very low latency is expected.

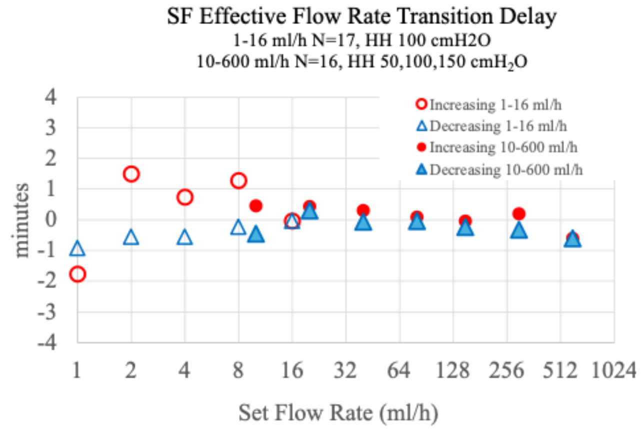

Fig. SM-6 Mean SF flow Transition Delays from 1 to 600 ml h-1 during increasing and decreasing flow steps.

D. Occlusion Detection Response Time
Occlusion detection times of SF and two LIPs were measured over a range of flow rates from 0.5 to 25 ml h^{-1} (Fig. SM-7). The LIPs were set to their low, mid and highest sensitivities, while SF requires no occlusion setting and was operated with 100 cmH_2O HH. LIPs using pressure measurement for occlusion detection have a large range of settings. For example, pump "B" has a low sensitivity (longest response) limit of 982 mmHg, while pump "A" has a high sensitivity (shortest response) at a setting of 75 mmHg.

Occlusion detection times for the LIPs demonstrated a kinematic relation to flow rate [17] given by:

$$TTO = P_{alarm} \cdot C_{tube} \cdot F_{set}^{-1} \qquad \text{(SM-3)}$$

where:
- TTO is time to detect alarm (minutes);
- P_{alarm} is the detection threshold (mmHg);
- C_{tube} is the tube compliance (ml $mmHg^{-1}$);
- F_{set} is the flow set flow rate (ml min^{-1}).

Data and resulting model fit equations in log-log and linear-log form are shown in Fig. SM-7.

SF produced shorter and significantly less variable occlusion times proportional to flow$^{-0.26}$, compared to LIPs whose occlusion times were inversely proportional to flow setting (~flow^{-1}). For the LIPs this produced rapidly increasing detection times at critical low flow rates under 1 ml h^{-1} but short response time at higher rates. The occlusion limit of the LIPs directly affected detection time while, for SF, direct flow measurement-based detection is independent of pressure.

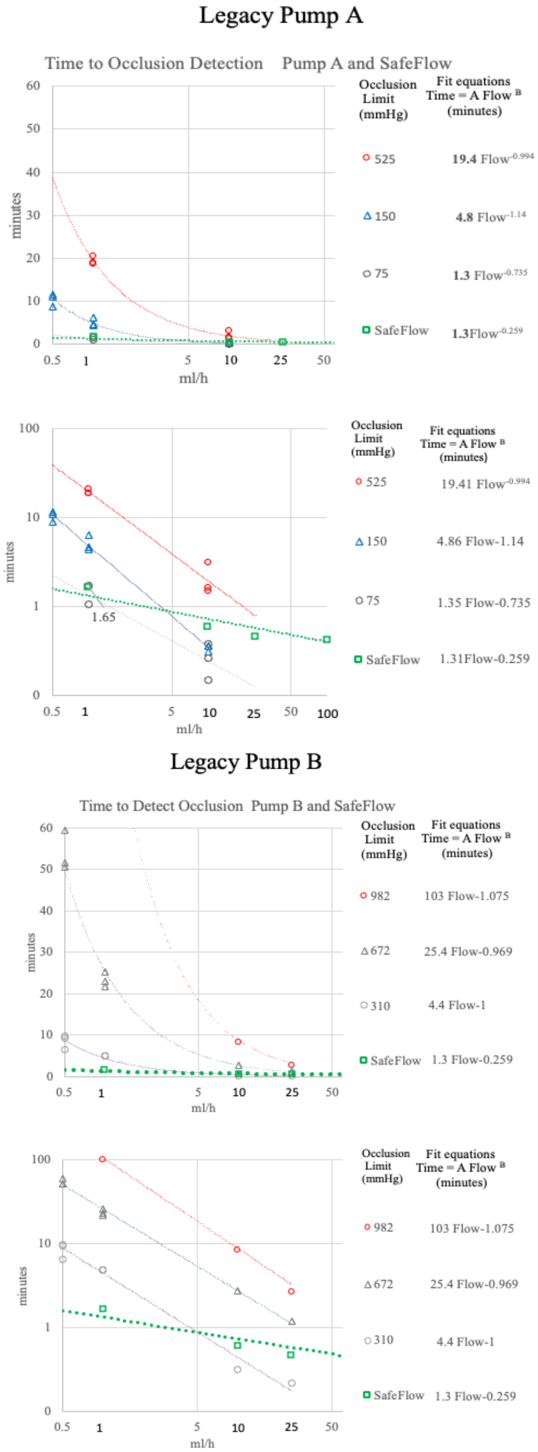

Fig. SM-7. Measured time (linear - top and log-bottom) to detect occlusion vs flow rate and limit settings for two LIPs vs SF.

E. Comparison with legacy technologies

Table SM-4 compares capabilities of SF with published and sample measurements of two widely used LIPs. These results are summarized in bar charts (see Fig. SM-8), showing SF vs LIP evaluations in the categories of: flow accuracy, sensitivity to HH, and flow variability, occlusion detection, maximum occlusion pressure, size, weight and battery run time.

**Characteristic**	**SF**	**LIP A**	**LIP B**
Flow Range (ml h^{-1})	1-600	0.1-999.9	0.5-999.9
Flow Rate Accuracy (Claimed, Measured)	+1.1% / -1.3% (mean range) -4.3 / 4.5 (95%/95% confidence/ reliability)	Claimed: ±5% "Standard Conditions" Measured: +1.5%	Claimed: ±5% DEHP tube +/-10% non-DEHP tube "Standard Conditions" Measured: +5.1%
Max Infusion Time (h)	72	96	96
Measured Sensitivity of MFRA to 0.5 m inlet head reduction	-0.85% (per 0.5m)	-2.5% (per 0.5m)	-4% (per 0.5m)
Measured Sensitivity of MFRA of 0.5 m outlet head increase		-0.72% (per 0.5m)	-1.2% (per 0.5m)
Measured Impact on MFRA of reduced inlet pressure allowed by device		-12.5% Flow Reduction	-17.6% Flow Reduction
Measured Impact MFRA of 10 psi outlet	N/A (occlusion detected at HH pressure)	-7.5% Flow Reduction	-12% Flow Reduction
Measured Short-Term Flow Accuracy (CV%) at1 ml h^{-1}	2.5 - 4%	4.5 - 10%	4 - 6%
Occlusion Time (mins) at 1 ml h^{-1} [threshold mmHg]	1.3	1.4, 4.9, 19 [75, 150, 525]	4.4, 25, 107 [310, 672, 982]
Maximum pressure (cmH_2O)	150	714	1224
Battery Life (h)	8 (claimed) 10 (max at 125 ml h^{-1})	4	4 (125 ml h^{-1})
Weight (kg)	0.45	4.95	1.625
Power Cord	OPTIONAL	YES	YES + A/C adapter

Table SM-IV. Performance and Feature comparison of SF with two widely-used LIPs.

IV. Discussion

A. Flow Range Capability

While the flow rate range of the SF is currently limited to 1-600 ml h^{-1}, adequate for a wide range of therapies, it may be extendable with addition of sets incorporating larger and smaller drip forming cannula and improved imaging capability for drop size estimation. Supply container switchover currently requires brief interruption of delivery. Under design is a set which will allow 'hot-swap' switching eliminating interruption.

B. Mean Flow Rate Accuracy (MFRA)

Compared to example LIPs, the SF devices achieved equivalent or better mean flow rate accuracy with significantly reduced susceptibility to elevation variation.

C. Short-term Flow Rate Variation (STFV)

SF's continuous propulsion, produced very low STFV at 1 ml h^{-1} (Fig. SM-5), well inside the CV range of 0-5% stated by TIR101 [11], without the mechanical flow pulsations exhibited by the LIPs. This characteristic may permit SF's use for administration of rapid-acting medications.

D. Flow Rate Change Response Time

Flow rate transition from stopped and between rates is essential for optimized titration and effect-site target control. Immediately following start, the SF devices produced a small advance in flow transition time, which typically is desirable in clinical scenarios where fast drug effect onset is desired. Since flow is controlled principally by the dynamics of the feedback control system , it may be possible to optimize transition as required for specific medications and use cases.

E. Occlusion Detection

At 1 ml h^{-1} , SF occlusion detection time of 1.3 minutes was at least as fast as LIP A set to its most sensitive threshold (rarely done in practice owing to risk of false alarms at higher flow rates). SF was 15 times faster than LIP-A and 79 times faster than LIP-B when these were each set to their least sensitive (highest pressure) occlusion detection settings.

In pressure-based occlusion detection used by legacy pumps, response time increases as flow rates decrease becoming unnecessarily fast at high flow rates. To avoid false alarms, clinicians often reduce occlusion sensitivity, with the unintended consequence of undesirably long detection time at low flow rates and potential large bolus release after occlusion release. [9] In contrast, SF occlusion detection time varied by only a factor of five over the range 1-600 ml h^{-1}, minimizing the need to adjust thresholds to obtain desired detection speed.

An unwanted byproduct of high pressures developed during occlusion in LIPs is the potential release of a stored bolus of fluid from an expanded delivery set. In the SF design, bolus release following release of an occlusion is inherently insignificant with the maximum pressure limited to ~100 cmH_2O due to head height. Bolus inadvertently infused after occlusion release was not measured for any of the devices under test, however manufacturer statements indicate that bolus release for LIPs can be as much as 0.6 ml at occlusion pressure limits as high as 982 mmHg.

F. Mobility and Bedside Organization for High Acuity Patients

A critical unsolved problem with high acuity patients is the large number of pumps required, mandating custom bedside and intra-hospital transport systems. [18] [19] [20] [21] [22] By virtue of its small size, advanced battery capacity, and ability to be remotely controlled by a smart phone or tablet, SF may alleviate this dilemma by providing a more ergonomic arrangement of devices on a single pole.

G. Remote-control/monitoring

The ability to monitor and control infusions, already implemented in SF's architecture (K213536, K241178 5/24/2024 and K233952 2/2024) [10] will provide benefits to daily workflows as well as for isolated patients, airborne infectious disease treatment, and patients undergoing hyperbaric oxygen treatment.

*H. Performance/Feature Compar*ison

Table SM-IV illustrates that SF achieved equivalent or superior performance to widely-used LIPs with a design providing inherent safety improvements related to superior fault detection and management, with reduced size/weight. Lower complexity and parts count may result in lower life cycle costs for end-users.

I. Potential enhancements

Example anticipated responses to end-user feedback and requests for use case extension:

- flow rate extension from 0.1 to 1 000 mL h^{-1}
- hot-swap containers via administration set extension above drip chamber
- protection from high downstream pressures (manual bolus) via custom manifold with check valve or limited motion stopcock

V. LIMITATIONS

While the SF device is equipped with a 3-axis accelerometer for use in avoiding false alarms during ambulation we did not test this capability.

We did not evaluate ability of SF to deliver boluses. Historically international standards have not included bolus testing for gravity-propulsion devices [12]. The SF platform is currently being evaluated for ability to deliver boluses of desired volumes and at chosen flow rates. Bolus administration rates similarly will likely be limited by fastest detectable flow rate.

Introduction of manual boluses in the patient line is common for both medication administration and flushing even though

clinicians are trained never to bolus into lines also carrying continuously titrated life-critical infusions. In some LIP's, a reflux of flow toward the pump may be produced. We did not test the SF devices response to this challenge. Mitigation using a mechanical forcing function in the form of a custom manifolds or check-valve is in evaluation.

We did not test electromagnetic compatibility (EMC, EMI) or susceptibility to static discharge.

VI. CONCLUSION

This study demonstrated feasibility of an infusion device combining machine-vision methods that enable continuous direct fluid flow measurement with gravitational propulsion.

Benefits of the SF design over LIP's include: increased reliability and durability through low mechanical parts-count, light-weight and small size, long-duration battery operation, minimum risk of medication container mix-up by elimination/minimization of supply-side tubing, faster and lower-risk occlusion detection without complex alarm threshold adjustments. These benefits are obtained currently with some reduction in maximum achievable flow rate range and limitation of bolus delivery ability - future enhancements are anticipated which will minimize these issues.

Referring to Table SM-IV and Fig. SM-8, we summarize key distinctions between LIPs and SF. Anticipated adoption challenges and opportunities await ongoing design evolution as exposure to real-world clinical settings suggests improvement. And, while future iterations may expand functionality, the currently available FDA-cleared device may be considered for applications within the scope of its capabilities.

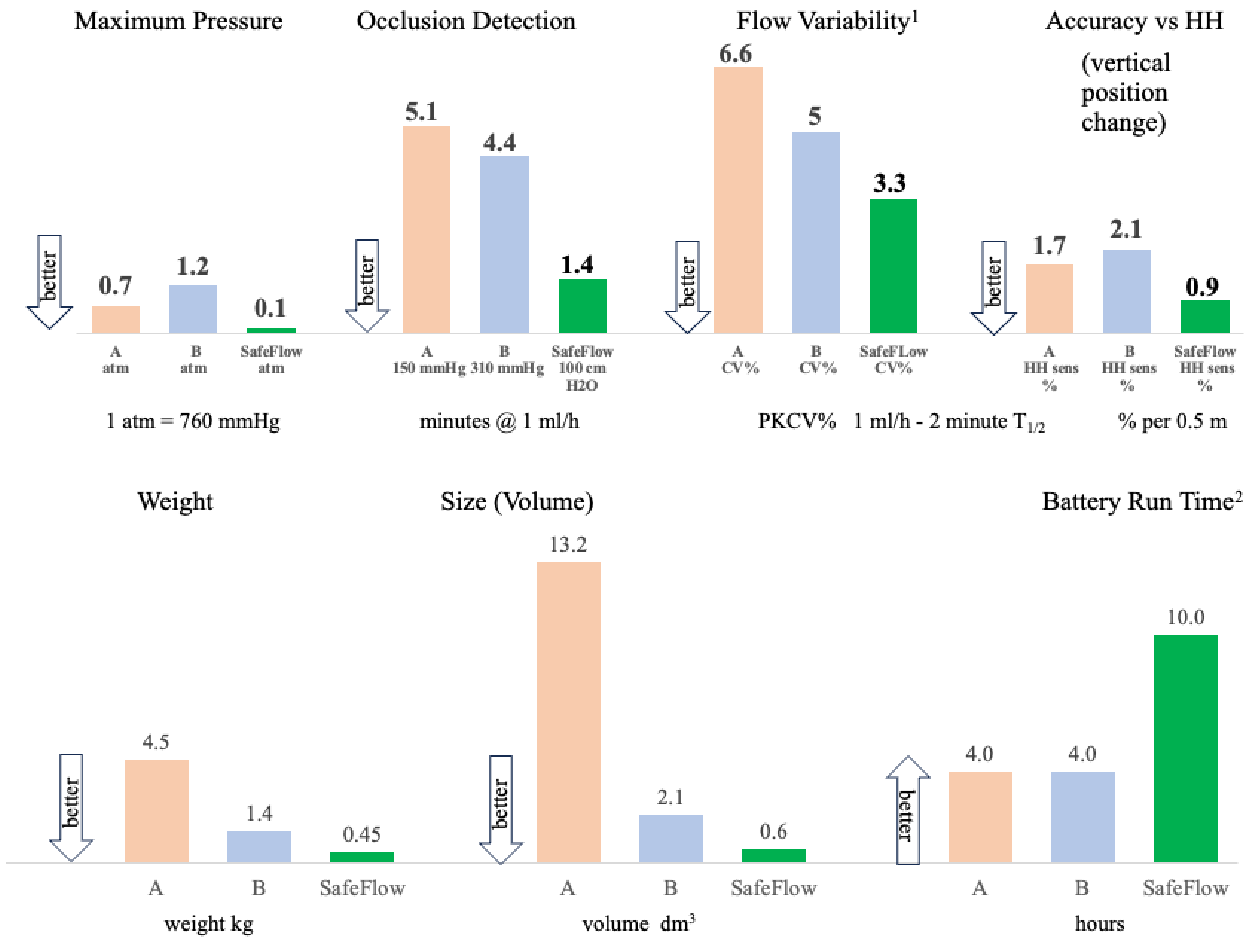

Fig. SM-8. Comparison of SF to LIP

APPENDICIES

A1. Flow Rate Range and Occlusion Detection Limitations

Currently the SF controllers' maximum flow rate is constrained to 600 ml h^{-1} both by optics and image processing and by drop forming orifice geometry.

Table SM-V shows the estimation of potential flow rate range and occlusion detection response time vs choice of drop-forming orifice. For example, use of a 10 gtts (drops per ml) orifice extends highest controllable flow rate but raises lowest flow which can be precisely controlled. The cleared SF design has a rate range of 10 to 300 ml h^{-1} while design enhancements in the evaluated SF, including direct monitoring of drop formation rate, extend rate range and shortened occlusion detection times.

Closed-loop control systems are inherently both benefitted and constrained by accuracy and resolution of flow sensing method applied. These permit adjustment for external variables to maintain target flow. Open loop systems, while able to provide a range of flows and fine discrete changes to flow control inputs, are unable to adapt when external pressures, temperatures and other variables impacting flow.

SIMULATOR - Dynamic Range Estimation vs Drip Orifice and Flow Rate

Drop Period Threshold

Fast Threshold(s)	0.2	More frequent drops can't be detected /resolved.
Slow Threshold(s)	10	Less frequent drops may compromise control speed during titration.
Set missed-drop threshold for occlusion →	1	

Steady State Flow

drops/ml	10	15	20	30	60	10	15	20	30	60
ul/drop	100	67	50	33	16.67					
set flow (ml/h)	Interval (sec/drop)					Estimated Occlusion Time (min)				
1	360	240	180	120	60	6.0	4.0	3.0	2.0	1.0
2	180	120	90	60	30	3.0	2.0	1.5	1.0	0.5
5	72	48	36	24	12	1.2	0.8	0.6	0.4	0.2
10	36.0	24.0	18.0	12.0	6.0	0.6	0.4	0.3	0.2	0.1
20	18.0	12.0	9.0	6.0	3.0	0.3	0.2	0.2	0.1	0.1
30	12.0	8.0	6.0	4.0	2.0	0.2	0.1	0.1	0.1	0.0
40	9.0	6.0	4.5	3.0	1.5	0.2	0.1	0.1	0.1	0.0
50	7.2	4.8	3.6	2.4	1.2	0.1	0.1	0.1	0.0	0.0
100	3.60	2.40	1.80	1.20	0.60	0.1	0.0	0.0	0.0	0.0
200	1.80	1.20	0.90	0.60	0.30	0.0	0.0	0.0	0.0	0.0
300	1.20	0.80	0.60	0.40	0.20	0.0	0.0	0.0	0.0	0.0
400	0.90	0.60	0.45	0.30	0.15	0.0	0.0	0.0	0.0	0.0
500	0.72	0.48	0.36	0.24	0.12	0.0	0.0	0.0	0.0	0.0
600	0.60	0.40	0.30	0.20	0.10	0.0	0.0	0.0	0.0	0.0
700	0.51	0.34	0.26	0.17	0.09	0.0	0.0	0.0	0.0	0.0
800	0.45	0.30	0.23	0.15	0.08	0.0	0.0	0.0	0.0	0.0
900	0.40	0.27	0.20	0.13	0.07	0.0	0.0	0.0	0.0	0.0
1000	0.36	0.24	0.18	0.12	0.06	0.0	0.0	0.0	0.0	0.0

FULL OPEN Gravity Flow. ** ISO 10,000 ml/h for wide open x 1m x DIW

Table SM-V Flow Rate and Occlusion Detection Estimation for a generic 'drop-counting' closed loop flow regulator. Pink cells denote either the response rate is too long for low rates or drip-rate is possibly too fast to be measured at highest flow rates. Right table shows projected occlusion detection time based on 'number of allowed missed drops' algorithm.

A2 .Transition Delay Calculations

Fig. SM-7 depicts how the lag time when transitioning between flow rates is computed using the "Start-of-Flow Delay method of AAMI TIR101 [11] was adapted. The volume-vs-time curve commencing at each subsequent flow is translated back to the origin converting raw V(nT) to V' simplifying calculation. The slope of the V'-time curve when in steady state (after T_{ss}) is computed as b and the intercept as a. These are in turn used to find the t_{delay} time illustrated by the thick double-ended arrow on timeline.

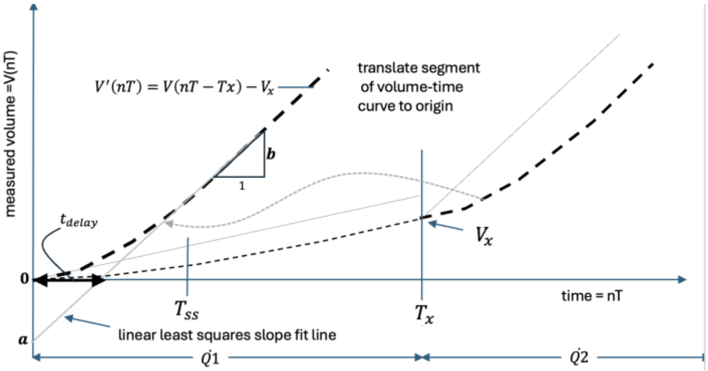

Fig. SM-9 Calculation of Transition Delay using adapted method of AAMI TIR101 [11]

$$t_{delay} = \frac{-\boldsymbol{a}}{\boldsymbol{b}} \quad \text{(SM-4)}$$

where:

$\dot{Q}x$ *denotes flow* $(ml\ h^{-1})$

$$V'(nT) = V(nT - Tx) - V_x;$$

$$\boldsymbol{a} = \frac{\sum_{i=l}^{i=u} V'_i - b\sum_{i=l}^{i=u} T'_i}{N_s};$$

$$\boldsymbol{b} = \frac{N_s \cdot \sum_{i=l}^{i=u}(V'_i \cdot T_i) - \sum_{i=l}^{i=u} T'_i \cdot \sum_{i=l}^{i=u} V'_i}{N_s \sum_{i=l}^{i=u} T'^2_i - (\sum_{i=l}^{i=u} T'_i)^2};$$

$$N_s = N_x - N_{ss};$$

$$u = \frac{T_x}{T};$$

$$l = \frac{T_{ss}}{T};$$

$$T = sample\ period\ (s).$$

A3. Numerical Simulation of Concurrent Flows

To model concurrent flow, where two or more SF flows merge through a common pathway, a numerical solution to the simultaneous equations of flow and resistance was implemented in LabView(R) (National Instruments / Emerson, Austin, Texas). This simulated behavior of infusion scenarios shown in Fig. SM-3b. A simplified version of the device's closed loop control method was used to adapt R1 and R2 control resistor values as H1, H2 head pressure and R3, the common fluid line catheter resistance, were varied. Use of the model assisted in selecting parameters for physical testing and exploring stability at boundary conditions.

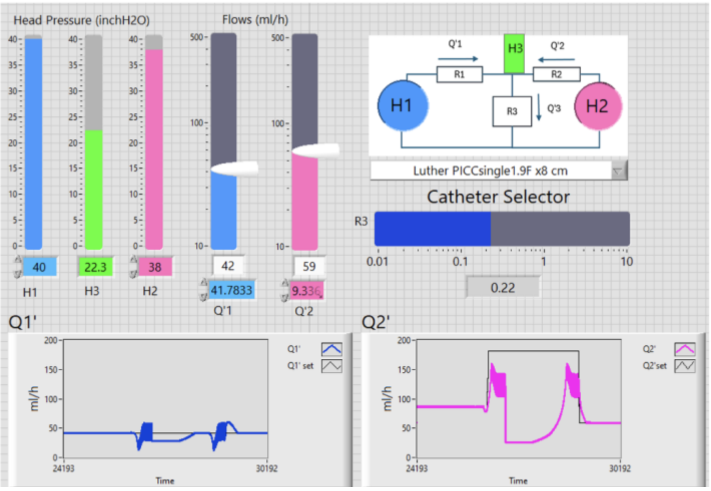

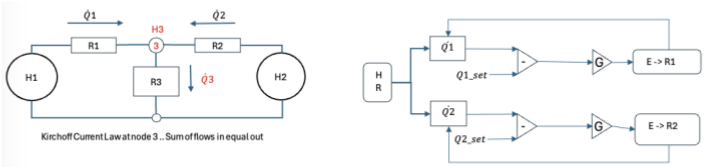

$$0 = -\dot{Q}1 - \dot{Q}2 + \dot{Q}3$$

$$0 = \frac{-(H1-H3)}{R1} - \frac{(H2-H3)}{R2} + \frac{H3}{R3}$$

$$0 = -\frac{H1}{R1} + \frac{H3}{R1} - \frac{H2}{R2} + \frac{H3}{R2} + \frac{H3}{R3}$$

$$\frac{H1}{R1} + \frac{H2}{R2} = \frac{H3}{R1} + \frac{H3}{R2} + \frac{H3}{R3}$$

$$\frac{H1}{R1} + \frac{H2}{R2} = H3\left(\frac{1}{R1} + \frac{1}{R2} + \frac{1}{R3}\right)$$

$$H3 = \frac{\frac{H1}{R1} + \frac{H2}{R2}}{\left(\frac{1}{R1} + \frac{1}{R2} + \frac{1}{R3}\right)}$$

$$\dot{Q}1 = \frac{(H1-H3)}{R3}$$

$$\dot{Q}2 = \frac{(H2-H3)}{R3}$$

$$\dot{Q}3 = \frac{H3}{R3}$$

Fig. SM-10a (top) Graphical control panel for numerical simulation of concurrent flow dynamics using LabView G-Language. SM-10b (bottom) equivalent circuit and G-Language implementation of simple feedback control loop simulating operation of actual SF controller.

REFERENCES

[1] M. Weinger and A. Kline, "Reflections on the Current State of Infusion Therapy," *Biomedical Instrumentation & Technology,* pp. 253-262, 2016.

[2] K. K. Giuliano, "IV Smart Pumps: The Impact of a Simplified User Interface on Clinical Use," *HORIZONS,* vol. Fall, pp. 13-21, 2015.

[3] BD, "Alaris™ System with Guardrails™ Suite MX (with Alaris™ PC unit, Model 8015 Software Version 9.33)," [Online]. Available: https://www.bd.com/content/dam/bd-assets/na/medication-management-solutions/documents/instructions-for-use/Alaris-System-8015.pdf.

[4] Baxter Healthcare, "https://infusystem.com/images/catalog_manuals/English/Pole_Mounted_Pump_Manuals/Spectrum-IQ-Operator-Manual-English-Rev-F-41018v0900.pdf," February 2020. [Online]. [Accessed 29 06 2025].

[5] J. W. Blake, R. Butterfield, T. Hopper and N. Sims, "Secondary Infusion Underdelivery: Risks and Rewards of Common Workarounds," *AACN Adv Crit Care,* vol. 36, no. 3, pp. 240-251, 15 September 2025.

[6] FDA, "AccessData.FDA.gov," [Online]. Available: https://www.accessdata.fda.gov/scripts/cdrh/cfdocs/cfmaude/search.cfm. [Accessed 2 06 2025].

[7] A. Lucchini, S. Elli, A. Burgazzi, L. Malvestuto Grilli, C. Pes, K. Ferrari, L. Fumagalli, C. Fiorillo, M. Giani and Emanuele Rezoagli, "Simulated haemodynamic parameters and different infusion set-up affect drug delivery during syringe pump change over: A bench-top study in a laboratory setting," *Intensiver & Critical Care Nursing,* vol. 86, pp. 1-7, 2025.

[8] A. Knudsen, "Pump-driven clinical infusions: laboratory comparison of pump types, fluid composition and flow rates on model drug delivery applying a new quantitative tool, the pharmacokinetic coefficient of short-term variation (PK-CV)," *J. Clin Monit Comput,* Vols. 10.1007/s10877-024-01200-y, 2024 (Sept 19).

[9] R. Ilan, "Prolonged time to alarm in infusion devices operated at low flow rates," *Critical Care Medicine,* vol. 36, no. 10, pp. 2763-2765, 2008.

[10] US FDA, "510(k) Clearances," [Online]. Available: https://www.fda.gov/medical-devices/device-approvals-and-clearances/510k-clearances. [Accessed 5 October 2025].

[11] AAMI, TIR101 Fluid Delivery Performance Testing for Infusion Pumps, Washington D.C.: Assn. for Advancement of Medical Instrumentation, 2021.

[12] IEC, IEC 60601-2-24 Medical electrical equipment Part 2-24: Particular requirements for the basic safety and essential performance of infusion pumps and controllers, Edition 2, vol. 2, International Electrotechnical Commission, 2012-10.

[13] "Neonatal PICC catheters Epicutaneo Cava (Si) 2fr / 24 G," [Online]. Available: https://www.vygon.com/en/products/vascular/neonatology-pediatrics/neonatal-picc-catheters/epicutaneo-cava-si-2fr-24-g.

[14] "Vascular Access Catalog - Arrow / Teleflex," [Online]. Available: https://www.teleflexvascular.com/products/ml-00703.

[15] "MedComp PICC Catheters," [Online]. Available: https://www.medcomp.net/PN2540_G_Vascu-PICC_Brochure_DOM.pdf#:~:text=Local%20tissue%20factors%20will%20prevent%20proper%20device,administration%20of%20fluids%2C%20medication%2C%20and%20nutritional%20therapy..

[16] D. A. Scott and J. H. Philip, "Resistance to Fluid Flow in Veins," *Journal of Clinical Monitoring,* vol. 12, pp. 331-337, 1996.

[17] R. D. Butterfield and G. V. Voss, "Chapter 85 Parenteral Infusion," in *Handbook of Biomedical Engineering*, CRC Press, 1995, p. 1319.

[18] ECRI, "ECRI 2017 Top Ten Hazards," 2017. [Online]. Available: https://www.ecri.org/resources/whitepapers_and_reports/haz17.pdf.

[19] M. G. Fitsimons and N. M. Sims, "Monitoring and Transport of the Critically Ill Patient," *Anesthesiology, 2e.,* no. Chapter 78, 2012.

[20] StreamLine IV, "SkyTron StreamLine IV," [Online]. Available: https://www.pic1medical.com/products/streamlineiv. [Accessed 7 5 2025].

[21] "Pic1Medical," [Online]. Available: https://www.pic1medical.com/categories/multipumpivtransportation. [Accessed 7 05 2025].

[22] J. H. Petre, "Patient equipment transport and support system". United States of America Patent 4901967.

[23] D. J. Berman, A. Schiavi, S. M. Frank, S. Duarte, D. A. Schwengel and C. R. Miller, "Factors that influence flow through intravascular catheters: the clinical relevance of Poiseuille's law," *Transfusion Medicine,* pp. 1410-1417, 2020.
